# Identification of Clock Genes Related to Hypertension in Kidney From Spontaneously Hypertensive Rats

**DOI:** 10.1093/ajh/hpaa123

**Published:** 2020-10-14

**Authors:** Yusuke Murata, Takahiro Ueno, Sho Tanaka, Hiroki Kobayashi, Masahiro Okamura, Seiichiro Hemmi, Yoshinobu Fuke, Yoshiaki Matsumoto, Masanori Abe, Noboru Fukuda

**Affiliations:** 1 Division of Nephrology, Hypertension and Endocrinology, Department of Internal Medicine, Nihon University School of Medicine, Tokyo, Japan; 2 Department of Clinical Pharmacokinetics, School of Pharmacy, Nihon University, Chiba, Japan; 3 Division of Cell Regeneration and Transplantation, Department of Functional Morphology, Nihon University School of Medicine, Tokyo, Japan

**Keywords:** blood pressure, clock gene, hypertension, kidney, nephrotubulus, spontaneously hypertensive rat

## Abstract

**Background:**

There is a diurnal variation in the blood pressure fluctuation of hypertension, and blood pressure fluctuation abnormality is considered to be an independent risk factor for organ damage including cardiovascular complications. In the current study, we tried to identify molecules responsible for blood pressure circadian rhythm formation under the control of the kidney biological clock in hypertension.

**Methods:**

DNA microarray analysis was performed in kidneys from 5-week-old spontaneously hypertensive rats (SHRs)/Izm, stroke-prone SHR rats (SHRSP)/Izm, and Wistar Kyoto (WKY)/Izm rats. To detect variation, mouse tubular epithelial cells (TCMK-1) were stimulated with dexamethasone. We performed immunostaining and western blot analysis in the renal medulla of kidney from 5-week-old WKY rats and SHRs.

**Results:**

We extracted 1,032 genes with E-box, a binding sequence for BMAL1 and CLOCK using a Gene Set Enrichment Analysis. In a microarray analysis, we identified 12 genes increased as more than 2-fold in the kidneys of SHRs and SHRSP in comparison to WKY rats. In a periodic regression analysis, phosphoribosyl pyrophosphate amidotransferase (Ppat) and fragile X mental retardation, autosomal homolog 1 (Fxr1) showed circadian rhythm. Immunocytochemistry revealed PPAT-positivity in nuclei and cytoplasm in the tubules, and FXR1-positivity in the cytoplasm of TCMK-1. In 5-week-old WKY rat and SHR kidneys, PPAT was localized in the nucleus and cytoplasm of the proximal and distal tubules, and FXR1 was localized to the cytoplasm of the proximal and distal tubules.

**Conclusions:**

PPAT and FXR1 are pivotal molecules in the control of blood pressure circadian rhythm by the kidney in hypertension.

Various biological phenomena, including the sleep cycle, heart rate, and blood pressure, have a circadian rhythm. For the circadian rhythm, the presence of biological clock was discovered, then it has been established that circadian rhythm exists in all living organisms from archaebacteria to human beings.^1^

The suprachiasmatic nucleus (SCN), which is considered to function as a central clock, is a center that exerts autonomous oscillation of circadian rhythm. In addition, internal clocks present in the peripheral organs are controlled by central clocks, and mark the appropriate rhythm for each organ.^2^ The central clock controls the peripheral clocks via neural and humoral factors. The SCN consists of a large number of neurons that oscillate autonomously, when these cells were isolated and dispersed in rats and mice, they showed circadian rhythms with slight differences in their firing frequency and gene expression. Thus, individual neurons in the SCN are clock cells with their own circadian oscillators.^3^

Recently, the molecular mechanisms underlying the rhythm of biological clocks have been clarified. The biological clock consists of a main loop in which brain and muscle arnt-like 1 (BMAL1), Circadian locomotor output cycles kaput (CLOCK), Period (PER), and Cryptochrome (CRY) work as core proteins. CLOCK and BMAL1 form a dimer to act as a transcription factor and bind to an E-box sequence (CACGTG).^4^ The target genes include other clock genes, such as Per and Cry. The generated PER and CRY proteins translocate into the nucleus and suppress the BMAL1 and CLOCK genes. Subsequently, PER and CRY are resolved over time through multistep phosphorylation by casein kinase (CKIε), glycogen synthase (GSK-3β), and AMP-activated protein kinase (AMPK). Thus, the suppression of BMAL1 and CLOCK is canceled by PER and CRY, and the promotion of the gene expression by CLOCK and BMAL1 starts again, and the clock goes around. In addition to this main loop, there is also a subloop involving nuclear receptor subfamily 1 group D member 1 (NR1D1), retinoic acid receptor-related orphan receptor (ROR), D-site of albumin promoter binding protein (DBP), and E4 promoter binding protein 4 (E4BP4). NR1D1 binds to the RRE (AGGTCA) sequence and suppresses the main loop via negative feedback, while DBP promotes the main loop via positive feedback by binding to the D-box (TTATGTAA) sequence.^5^ It is also reported that proteins ticking the circadian rhythm under the control of biological clock reach approximately 15% in each tissue.^6^ The biological clock is involved in the formation of circadian rhythms of various physiological phenomena.

It is known that there is a diurnal variation in the blood pressure fluctuation of hypertension, and blood pressure fluctuation abnormality is considered to be an independent risk factor for organ damage including cardiovascular complications.^7^ Disordered blood pressure circadian rhythm causes organ damage. There are many reports on the relationship between blood pressure and biological clocks, such as Per, Cry, Bmal1, and Clock. Decreased blood pressure, loss of daily blood pressure fluctuation, and vascular endothelial cell injury have been reported in Bmal1 knockout mice.^8^ Clock knockout mice have reported to show increased urinary sodium excretion, decreased blood pressure, and even a reduced heart rate and decreased heart rate circadian amplitude.^9^ Dashti *et al.* reported that the clock genes induce a large proportion of phenotypic variance in systolic blood pressure.^10^

Regarding the contribution of the kidneys to the circadian rhythms, normal kidneys have greater electrolyte excretion and urine production during the day than at night, and there is a circadian rhythm in sodium, potassium, and chlorine excretion.^11^ In hypertension, failure of the circadian rhythm of these functions is reported to be associated with cardiovascular complications.^12^ Per1 knockout mice show increased sodium excretion and decreased blood pressure in relation to the α subunit of the renal epithelial sodium channel (αENaC) in the collecting duct of the kidney, indicating the presence of a biological clock in the kidney.^13^ In addition, diurnal variation in blood pressure (nondipper status) is observed in patients with chronic kidney disease.^14^ Thus, the central clock located in the SCN regulates the renal peripheral clock, marking the optimal timing for the kidney.

The spontaneously hypertensive rat (SHR), a genetic model of essential hypertension, has been reported to show deviation in heart rate and diurnal fluctuation of blood pressure, which are enhanced by aging.^15^ Previously, we investigated the adrenal gland circadian clock in SHR and Wistar Kyoto (WKY) rats maintained under a 12-hour light–dark cycle and found that SHR possess an abnormal adrenal circadian clock and show the altered circadian rhythm of clock-controlled gene coding rate-limiting enzyme in steroid genesis as well as altered serum glucocorticoid level.^16^ In addition to the signal from the central SCN clock, glucocorticoid released from the adrenal gland is known to synchronize peripheral circadian clock in other organ.^17^ Dexamethasone stimulation can reproduce the circadian fluctuation of clock gene and be applicable for *in vitro* study.^18^ In this circumstance, it is possible that SHR also has abnormal peripheral circadian clocks in various organs including kidney which plays a pivotal role in blood pressure regulation. Nevertheless, circadian clock in kidney from SHR has not been fully understood.

In the current study, we tried to identify the responsible clock genes of the kidney in hypertension using SHR and stroke-prone SHR rats (SHRSP), and evaluated the roles of the clock genes involved in the regulation of the abnormal blood pressure circadian rhythm.

## METHODS

### Ethics and animals

Our investigation conformed to the Guide for the Care and Use of Laboratory Animals published by the US National Institutes of Health (NIH Publication No. 85-23, 1996). Research protocols involving the use of living animals were assessed by the ethics committee of the Nihon University School of Medicine (No. 11–034). WKY/Izm rats and SHR/Izm maintained at Disease Model Cooperative Research Association were purchased from Japan SLC (Hamamatsu, Shizuoka, Japan). Systolic blood pressure was measured by the tail-cuff method. In this study, in order to avoid secondary changes in gene expression by the elevation of blood pressure and to identify responsible genes that affect circadian rhythms and eventually associate hypertension, we chose prehypertensive 5-week-old rats.

### Search for genes with E-box in whole genome

We used the Molecular Signatures Database in Gene Set Enrichment Analysis (GSEA; Broad Institute, Boston, MA) to search the gene cluster for human transcription factor targets and used the database with the highest number of genes upstream from the transcription start point A gene, in which E-box (CACGTG) was present at 2,000 bp upstream.

### DNA microarray analysis

Kidneys were removed from 5-week-old SHR/Izm, SHRSP/Izm, and WKY/Izm rats at 12:00. The resected kidney was homogenized with a TaKaRa BioMasher Standard (Takara, Otsu, Shiga, Japan), and total RNA was extracted. After hybridization and scanning, quantification was performed with Agilent Feature Extraction 10.7.3.1 (Agilent Technologies). The gene expression was compared and examined. Genes whose expression fluctuates between strains were extracted with a threshold of more than 2-fold or less than 0.5-fold.

### Detection of variation of candidate genes in TCMK-1 cells

Mouse tubular epithelial cells (TCMK-1) from American Type Culture Collection (ATCC, Manassas, VA) were stimulated with 0.5 µmol/l dexamethasone as described previously.^18^ The medium was changed to serum-free Dulbecco’s Modified Eagle Medium. Total RNA was extracted every 4 hours after dexamethasone stimulation, and the abundance of Npm1, Nptx1, Trim46, Plbd1, Plagl1, Bcl6, Fxr1, Tbl1, Hnrnpa3, Ppat, and Tef mRNAs was quantified using real-time polymerase chain reaction. At the same step, real-time polymerase chain reaction was performed on glyceraldehyde 3-phosphate dehydrogenase (GAPDH) RNA and used as an internal standard. The primers used are listed in Supplementary Table S1 online.

The periodicity of mRNA fluctuation after dexamethasone stimulation of TCMK-1 cells was analyzed by a multivariate regression analysis that determined the mesh curve (MESS), amplitude (AMP), phase (TZ1) by optimizing the cosine curve using the least squares method Estimates of MESS, AMP, and TZ1; their 95% confidence intervals were calculated using the single-coincidence method.

### Immunofluorescent staining of TCMK-1 cells

TCMK-1 cells were fixed with 4% paraformaldehyde. After washing with phosphate buffered saline, cells were incubated with rabbit monoclonal anti-PPAT antibody (Sigma-Aldrich) diluted 250 times and rabbit monoclonal anti-FXR1 antibody (Abcam, Cambridge, UK) diluted 300 times at room temperature for 1 hour. A 1,000-fold dilution of Alexa Fluor 488 anti-rabbit IgG (Invitrogen), as a secondary antibody, was made to react in the dark for 1 hour.

### Immunostaining in the kidney from WKY rats and SHR

The renal medulla of the kidney from 5-week-old WKY/Izm rats and SHR/Izm was isolated and embedded in paraffin, and incubated with rabbit monoclonal anti-PPAT antibody diluted 250 times, and rabbit monoclonal anti-FXR1 antibody diluted 200 times. Histofine Simple Stain Rat MAX-PO (MULTI) (Nichirei Biosciences, Tokyo, Japan) was applied as a secondary antibody. The slices were dehydrated, cleared, sealed with marinol, and observed under an optical microscope.

### Western blotting for FXR1 and PPAT proteins in the kidneys from WKY rats and SHR

The renal medulla was subjected to protein extraction using radio-immunoprecipitation assay buffer (Nacalai Tesque, Kyoto, Japan). The slices were transferred to a polyvinylidene difluoride membrane with iblot (Invitrogen) and blocked with 5% skimmed milk solution for 1 hour. The primary antibodies, which were rabbit monoclonal anti-FXR1 antibody, and rabbit monoclonal anti-PPAT antibody were applied. Horseradish peroxidase-conjugated goat anti-rabbit IgG (Jackson ImmuneReseach, West Grove, PA) was used as a secondary antibody. GAPDH was used as internal standards.

### Statistical analyses

The values were reported as the mean ± SE. Student’s *t*-test was used for the analysis of unpaired data. A 2-way analysis of variance with the Bonferroni/Dunn procedure as a post-test was also used. *P* values of <0.05 were considered to indicate statistical significance.

## RESULTS

### Blood pressure

The systolic blood pressure of 5-week-old WKY/Izm rats, SHR/Izm rats, and SHRSP/Izm rats was 116 ± 3.0 (*n* = 6), 121 ± 3.3 (*n* = 6), and 125 ± 3.4 (*n* = 5) mm Hg, respectively. The systolic blood pressure did not differ among the 3 strains to a statistically significant extent.

### Search for genes with E-box in promoter by GSEA analysis

We searched for genes carrying the E-box (CACGTG) binding sequence of CLOCK and BMAL1 heterodimer in the entire genome. As a result, 1,032 genes carrying an E-box between 2,000 bp upstream or downstream from the transcription initiation point were identified (Supplementary Table S2 online).

### Comparison of the gene expression in the kidney from WKY, SHR, and SHRSP rats by DNA microarray analysis

We compared the gene expression in kidneys from 5-week-old WKY/Izm rats and SHR/Izm rats using DNA microarrays. Three hundred sixty-three genes were highly expressed (more than 2-fold) in the renal cortex of SHR/Izm rats in comparison to WKY/Izm rats. The expression levels of 281 genes in the kidney were more than 0.5-fold in comparison to WKY/Izm rats. In the renal medulla of SHR/Izm rats, 284 genes showed higher expression levels and 203 genes showed lower expression levels in comparison to WKY/Izm rats. In the renal cortex of SHRSP/Izm rats, the expression levels of 267 genes were more than 2-fold in comparison to those in WKY/Izm rats. The expression levels of 580 genes were less than 0.5-fold in comparison to WKY/Izm rats. In the renal medulla of SHRSP/Izm, 639 genes showed higher expression levels and 251 genes showed lower expression levels in comparison to WKY/Izm rats. Among 1,032 genes with an E-box, the expression level of 17 gene mRNAs was more than 2-fold in the renal cortex of SHR/Izm rats in comparison to WKY/Izm rats. Among these 17 genes, the expression of 4 genes (Bcl6, Nr1d1, Ppat, and Tef) was increased more than 2-fold in the renal cortex of SHRSP/Izm rats in comparison to WKY/Izm rats (Table 1). In the renal cortex of SHR/Izm rats, expression of 10 genes mRNA was detected, which were decreased as less than 0.5-fold in comparison to WKY/Izm rats; 8 of these genes (Fxr1, Hnrnpa3, Npm1, Nptx1, Plagl1, Plbd1, Tbl1x, and Trim46) were also decreased as less than 0.5-fold in the renal cortex of SHRSP/Izm rats (Table 1).

**Table 1.** Comparison of the gene expression in the kidney from WKY rats, SHR, and SHRSP by DNA microarray analysis

		WKY rats			SHR			SHRSP		
Gene name	Description	Normalized	Raw	Fold change	Normalized	Raw	Fold change	Normalized	Raw	Fold change
SHR/WKY rats >2.0 in renal cortex										
Bcl6	B-cell CLL/lymphoma 6	0.87	95.6	1	2.73	253.5	3.13	2.80	207.2	3.21
Dnajb9	DnaJ (Hsp40) homolog, subfamily B, member 9	0.21	18.9	1	0.47	37.8	2.25	0.49	34.4	1.58
Fkbp5	FK506 binding protein 5	0.79	73.1	1	4.00	309.0	5.09	1.09	84.8	1.30
Gmfb	Glia maturation factor, beta	0.18	16.4	1	0.38	29.2	2.14	0.32	22.7	1.13
Gucy1a2	Guanylate cyclase 1, soluble, alpha 2	0.14	20.0	1	0.331	25.4	2.46	0.20	13.2	1.51
Lztfl1	Leucine zipper transcription factor-like 1	0.11	12.2	1	0.348	26.6	3.13	0.13	10.4	1.20
Nr1d1	Nuclear receptor subfamily 1, group D, member 1	0.07	6.13	1	0.443	35.3	6.71	0.43	29.9	6.48
Pdia4	Protein disulfide isomerase family A, member 4	0.73	65.5	1	1.6	122.4	2.18	0.96	64.3	1.31
Ppargc1b	Peroxisome proliferator-activated receptor gamma, coactivator 1 beta	1.05	97.0	1	2.80	214.2	2.66	1.74	118.9	1.65
Ppat	Phosphoribosyl pyrophosphate amidotransferase	0.33	30.3	1	0.73	55.9	2.19	0.81	54.4	2.44
Slc39a11	Solute carrier family 39 (metal ion transporter), member 11	0.16	14.2	1	0.34	25.7	2.15	0.270	18.3	1.71
Slc43a1	Solute carrier family 43, member 1	0.12	12.7	1	0.39	31.1	3.32	0.24	16.4	1.21
Tef	Thyrotrophic embryonic factor	0.20	18.1	1	1.03	82.8	5.27	0.53	37.5	2.57
Tesk2	Testis-specific kinase 2	0.17	15.7	1	0.39	29.8	2.27	0.12	8.91	0.71
Tgfb2	Transforming growth factor, beta 2	0.09	10.5	1	0.34	26.2	3.59	0.07	4.67	1.28
Tsku	Tsukushi small leucine rich proteoglycan homolog (*Xenopus laevis*)	4.68	429.0	1	10.2	791.9	2.17	6.02	409.2	1.28
Zmym6	Zinc finger, MYM-type 6 (Zmym6), mRNA	0.16	14.4	1	0.32	24.9	2.03	0.19	13.1	0.79
SHR/WKY rats <0.5 in renal cortex										
Anapc13	Anaphase promoting complex subunit 13	0.60	58.8	1	0.22	22.3	0.37	0.42	30.9	1.17
Fxr1	Fragile X mental retardation, autosomal homolog 1	0.59	55.1	1	0.22	20.6	0.38	0.29	21.6	0.49
Hnrrnpa3	Heterogeneous nuclear ribonucleoprotein A3	0.64	73.8	1	0.31	28.1	0.49	0.22	19.0	0.35
Npm1	Nucleophosmin (nucleolar phosphoprotein B23, numatrin)	1.98	238.0	1	0.96	87.4	0.49	1.03	73.8	0.50
Nptx1	Neuronal pentraxin 1	0.49	43.6	1	0.051	3.87	0.10	0.08	5.08	0.18
Plagl1	Pleiomorphic adenoma gene-like 1	0.53	47.8	1	0.26	20.5	0.50	0.20	13.3	0.40
Plbd1	Phospholipase B domain containing 1	0.32	29.8	1	0.14	14.1	0.44	0.12	9.17	0.38
Tbl1x	Transducin (beta)-like 1 X-linked	0.64	66.7	1	0.30	36.1	0.48	0.32	22.0	0.50
Trim46	Tripartite motif-containing 46	0.42	38.7	1	0.10	7.85	0.23	0.20	14.6	0.48
Tfrc	Transferrin receptor	0.81	83.6	1	0.30	28.1	0.37	0.79	52.8	2.14
SHR/WKY rats >2.0 in renal medulla										
Dnajb9	DnaJ (Hsp40) homolog, subfamily B, member 9	0.21	18.4	1	0.46	37.29	2.20	0.37	31.7	1.60
Fkbp5	FK506 binding protein 5	0.59	54.2	1	2.50	206.2	4.24	1.20	106.0	1.90
Nr1d1	Nuclear receptor subfamily 1, group D, member 1	0.21	18.3	1	0.65	55.0	3.13	0.44	38.6	2.10
Nrip3	Nuclear receptor interacting protein 3	0.17	22.7	1	0.44	37.4	2.57	0.56	57.0	0.97
Ppargc1b	Peroxisome proliferator-activated receptor gamma, coactivator 1 beta	1.13	102.2	1	2.71	225.8	2.40	1.63	138.0	1.44
Pwp2	PWP2 periodic tryptophan protein homolog (yeast)	0.61	52.7	1	1.26	105.5	2.08	0.17	17.8	0.80
Slc38a2	Solute carrier family 38, member 2	1.21	104.6	1	2.54	224.6	2.11	1.55	133.2	1.33
Tef	Thyrotrophic embryonic factor	0.27	27.8	1	0.83	68.0	3.01	0.73	65.1	2.52
Tnfrsf21	Tumor necrosis factor receptor superfamily, member 21	0.32	31.4	1	0.84	68.8	2.63	8.26	704.2	1.37
Tsku	Tsukushi small leucine rich proteoglycan homolog (*Xenopus laevis*)	3.38	306.1	1	7.66	638.8	2.27	3.33	287.3	0.99
SHR/WKY rats <0.5 in renal medulla										
Tfrc	Transferrin receptor	0.80	69.1	1	0.35	35.7	0.44	0.63	53.4	2.76

The mRNA expression of 10 genes in the renal medulla from SHR/Izm rats was increased more than 2-fold in comparison to WKY/Izm rats. Among these 10 genes, the Nr1d1 and Tef mRNA expression in the renal medulla from SHRSP/Izm rats was increased more than 2-fold in comparison to WKY/Izm rats (Table 1). The Transferrin receptor (Tfrc) mRNA expression in the renal medulla of SHR/Izm rats showed as less than 0.5-fold in comparison to WKY/Izm rats. However, the Tfrc mRNA expression was increased in the renal medulla of SHRSP/Izm rats (Table 1).

### Induction of clock gene mRNAs in nephrotubular cells

**Figure 1.**
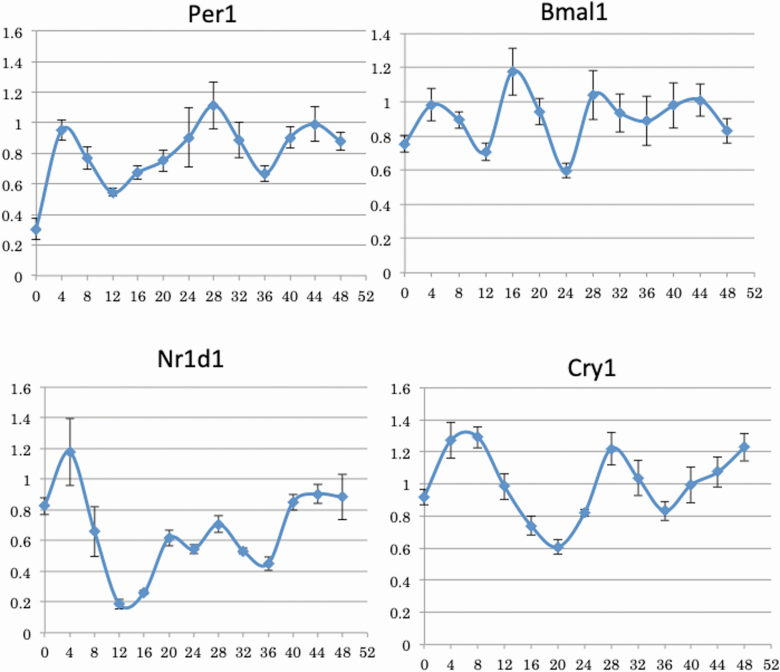
The expression variation of clock gene mRNAs in TCMK-1 cells after dexamethasone stimulation. TCMK-1 cells were stimulated with 0.5 µmol/l dexamethasone for 2 hours. Total RNA was extracted every 4 hours after dexamethasone stimulation, and the abundance of Per1, Bmal1, Nr1d1, and Cry1 mRNAs was evaluated by real-time polymerase chain reaction. The relative gene expression was analyzed by comparison to GAPDH mRNA. Data are presented as the mean ± SEM (*n* = 6).

**Figure 2.**
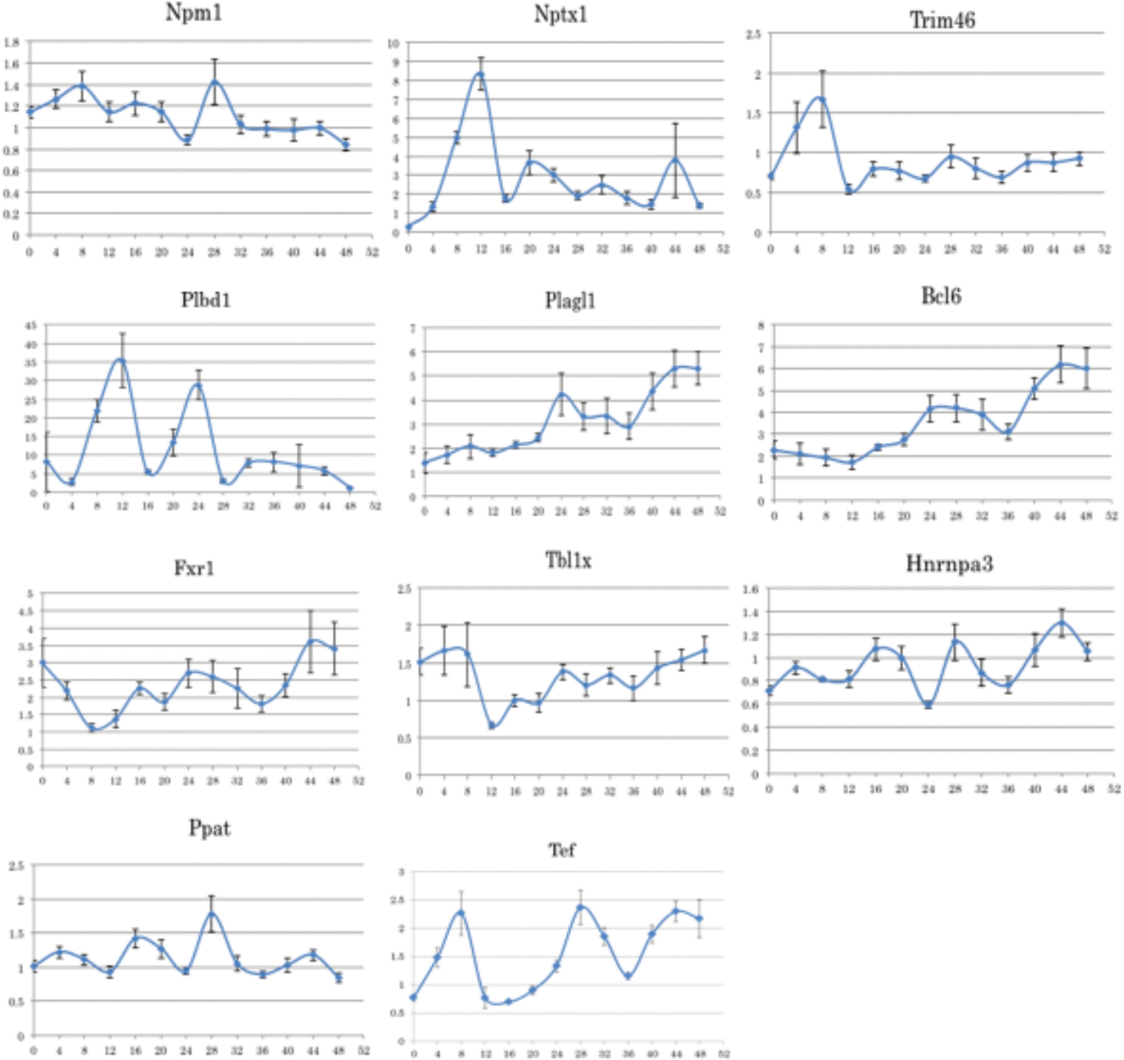
The expression variation of candidate gene mRNAs in TCMK-1 cells after dexamethasone stimulation. TCMK-1 cells were stimulated with 0.5 µmol/l dexamethasone for 2 hours. Total RNA was extracted every 4 hours after dexamethasone stimulation, and the abundance of Npm1, Nptx1, Trim46, Plbd1, Plagl1, Bcl6, Fxr1, Tbl1, Hnrnpa3, Ppat, and Tef mRNAs was evaluated by real-time polymerase chain reaction. The relative gene expression was analyzed by comparison to GAPDH mRNA. Data are presented as the mean ± SEM (*n* = 6).

Figure 1 shows the variation in the expression of Per1, Bmal1, Nr1d1, and Cry1 mRNAs after the addition of dexamethasone in TCMK-1 cells. The amplitudes of the Per1, Bmal1, and Nr1d1 mRNA expression showed peaks at 28 and 44 hours after 4 hours of dexamethasone stimulation. The amplitude of the Cry1 mRNA expression showed peaks at 4, 28, and 44 hours after 4 hours of dexamethasone stimulation. Thus, dexamethasone stimulation induced the fluctuation of clock gene mRNA expression in TCMK-1 (Figures 1 and 2).

### Investigation of candidate genes according to the mRNA expression with dexamethasone stimulation

We evaluated the induction of expression amplitude of 11 genes (excluding Nr1d1) in TCMK-1 cells after dexamethasone stimulation. The mRNA expression of Ppat peaked at 28 hours after stimulation with dexamethasone, and then increased again at 44 hours. Hnrnpa3 showed peaks at 16, 28, and 44 hours and showed amplitudes relatively similar to Per1. Npm1 showed a peak at 28 hours, similar to Per1. Fxr1 showed a peak at 24 and 44 hours (Figure 2).

We performed a periodic regression analysis to evaluate the statistical significance of these variations. The analysis indicated that the variation of the Cry1 and Bmal1 mRNA expression showed significant (*P* < 0.05) periodicity after dexamethasone loading in TCMK-1 cells (Supplementary Figure S1 and Table S3 online). The results of periodic regression analysis of 7 genes are shown in Supplementary Figure S2 and Table S3 online. The analysis indicated that Fxr1 and Ppat showed significant (*P* < 0.05) regression. In the regression of Fxr1, both the period and amplitude showed large waves, while in Ppat, both the period and amplitude were small and showed ultradian rhythm.

### Immunostaining of PPAT and FXR1 in nephrotubular cells and in kidneys from WKY and SHR rats

**Figure 3.**
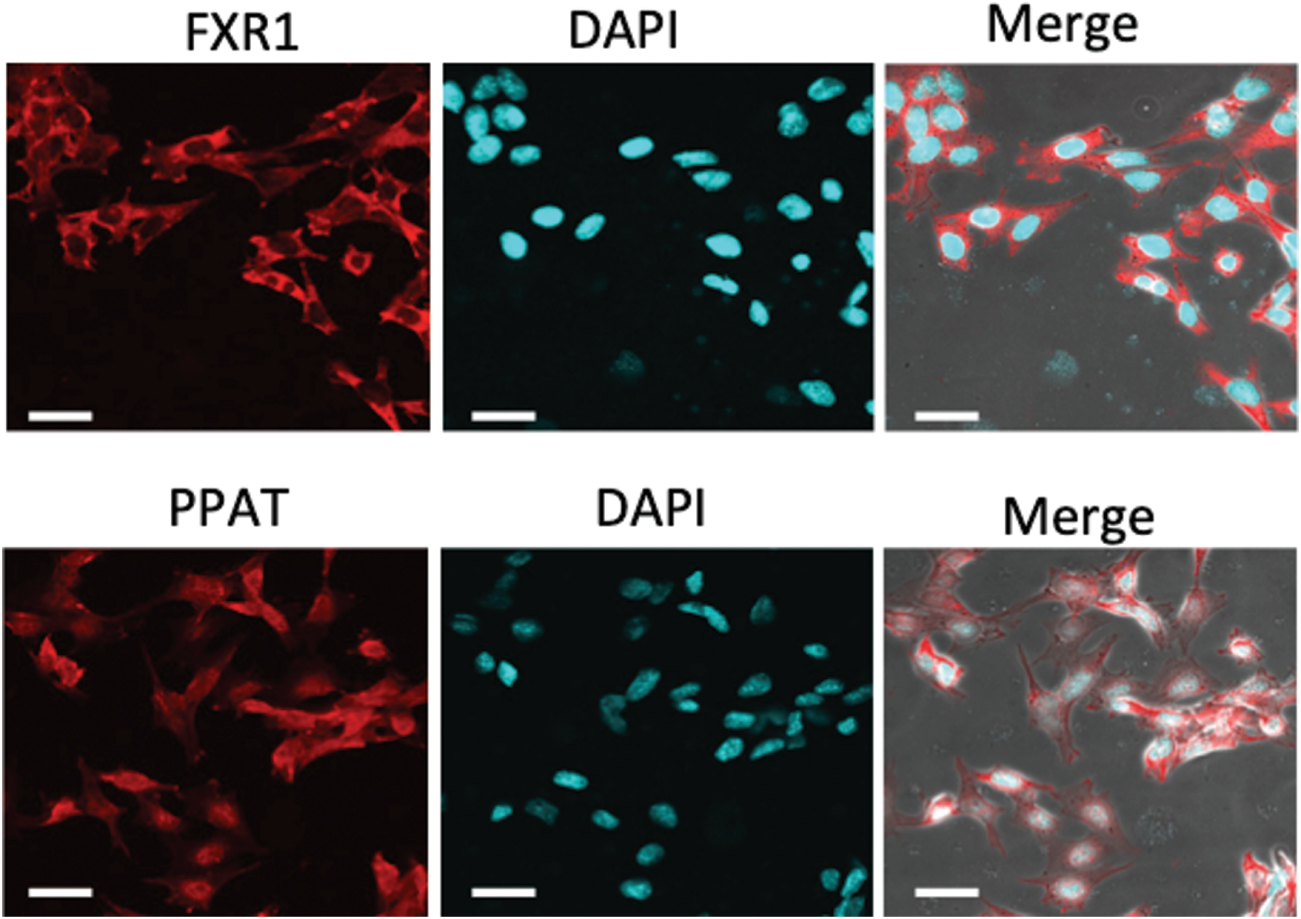
Immunocytochemical staining of FXR1 and PPAT in TCMK-1 cells. TCMK-1 cells were stained with anti-PPAT and anti-FXR1 antibodies. Nuclei were stained with Hoechst 33342 (4’,6-Diamidino-2-phenylindole). Scale bar = 25 µm.

**Figure 4.**
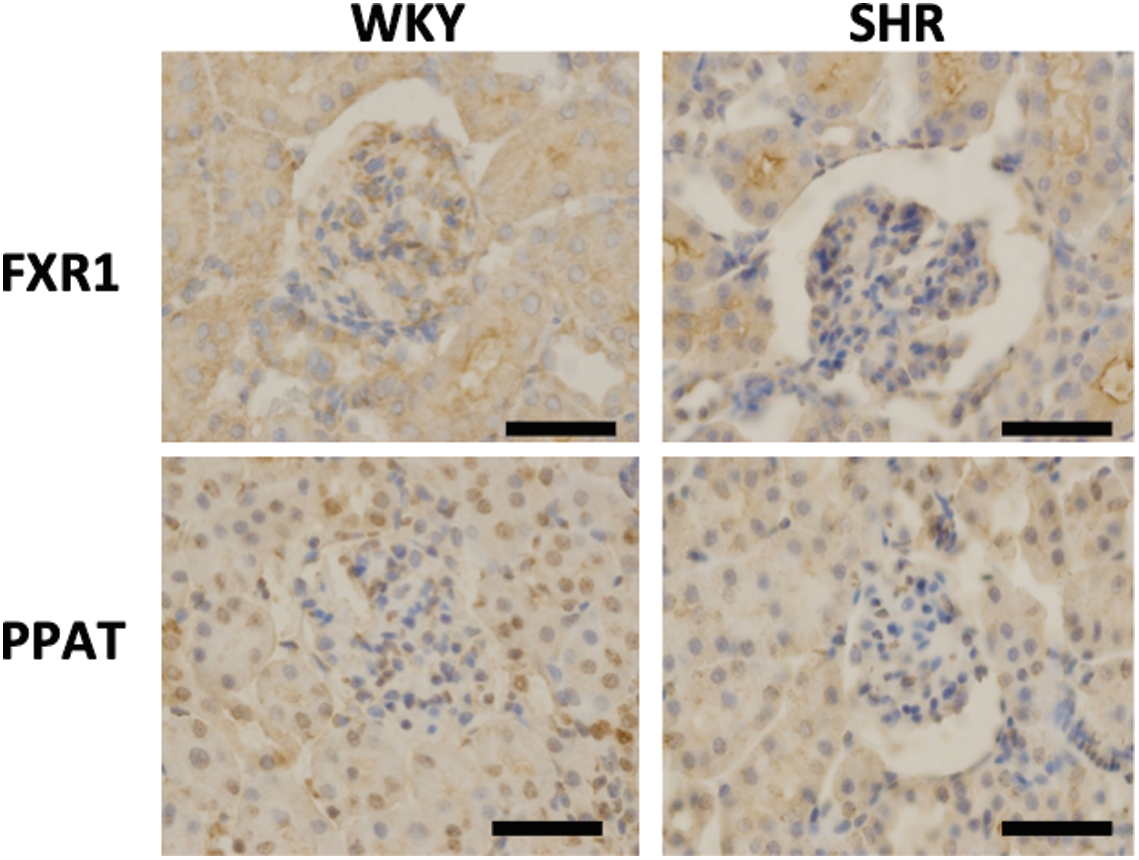
Immunohistochemical staining of FXR1 and PPAT in the renal cortex of kidneys from 5-week-old Wistar Kyoto (WKY) rats and spontaneously hypertensive rats (SHRs). Scale bar = 25 µm. Original magnification ×200.

PPAT was localized in both the cytoplasm and nucleus. FXR1 was only localized in the cytoplasm of TCMK-1 cells (Figure 3). PPAT was stained in both the nuclei and cytoplasm in the proximal and distal tubules (Figure 4). FXR1 was mainly stained in the cytoplasm of the proximal and distal tubules (Figure 4). There were no apparent differences in staining or localization in the kidneys of WKY and SHR rats.

### The FXR1 and PPAT protein expression in kidneys from WKY and SHR rats

**Figure 5.**
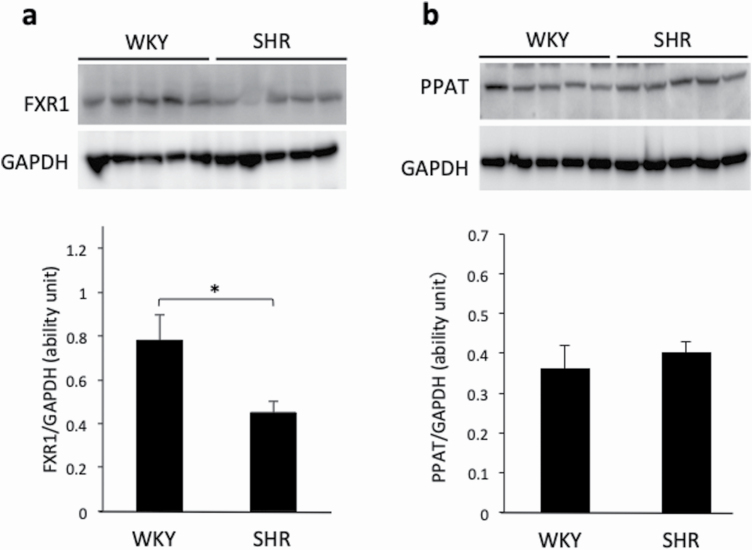
Western blotting of FXR1 (**a**) and PPAT (**b**) in the renal medulla of 5-week-old Wistar Kyoto (WKY) rats and spontaneously hypertensive rats (SHRs). Data are presented as the mean ± SEM (*n* = 5). *P < 0.05 between the indicated columns.

Figure 5 shows the western blotting of FXR1 and PPAT proteins in the renal medulla from 5-week-old WKY and SHR rats. The abundance of FXR1 protein was in the renal medulla of SHR rats was significantly lower in comparison to WKY rats (*P* < 0.05). The PPAT protein expression in the renal medulla of WKY and SHR rats did not differ to a statistically significant extent.

## Discussion

Hypertension induces cardiovascular and renal complications, such as chronic kidney disease (CKD). Patients with CKD are known to develop blood pressure fluctuation abnormalities, suggesting that the kidney may be involved in the abnormal blood pressure circadian rhythms in hypertension.^19^ In the current study, we therefore aimed to identify molecules responsible for blood pressure circadian rhythm formation under the control of the biological clock of the kidney in hypertension.

We obtained 1,032 candidate genes carrying the E-box sequence from the GSEA database. Because BMAL1/CLOCK heterodimer binds E-box to regulate downstream gene transcription, these genes are considered to be controlled by a biological circadian clock. In order to identify molecules involved in the blood pressure circadian rhythm in the kidney, we comprehensively investigated the gene expression in kidneys from SHR, SHRSP, and WKY rats using a DNA microarray. Among the 1,032 genes, we selected transcripts that increased more than 2-fold or decreased less than 0.5-fold in SHR kidney relative to those of WKY rats. We then focused on genes that showed similar trends in the kidneys of SHRSP, specifically, *Bcl6*, *Nr1d1*, *Ppat*, *Tef*, *Fxr1*, *Hnrnpa3*, *Npm1*, *Nptx1*, *Plagl1*, *Plbd1*, *Tbl1x*, and *Trim46*. These 12 genes carrying the E-box sequence, which showed higher or lower expression levels in both hypertensive model rats (SHR and SHRSP) in comparison to normotensive WKY rats, are possibly associated with the blood pressure circadian rhythm. Among these genes, *Nr1d1* also called Reverse erythroblastosis virus-α (*Rev-erbα*), has been reported to form a subloop of the biological clock.^20^

It has been confirmed that subjecting cultured cells to dexamethasone loading or high-concentration horse serum shock reproduced the amplitude of the clock gene expression *in vitro*.^21,22^ The addition of high-concentration serum and glucocorticoid are considered to be useful for the reproduction of the circadian rhythm of the clock gene expression in peripheral tissues.^18,23^ In this study, we succeeded in inducing the amplitude of the Per1, Cry1, and Nr1d1 mRNA clock genes *in vivo* with dexamethasone loading in mouse tubular epithelial cells. With dexamethasone loading, Per1 was increased at 4, 20–24, and 44 hours after stimulation, Cry1 was increased at 0–8 and 24–32 hours, and Nr1 d1 was increased at 0–4, 16–24, and 40–44 hours. These changes are similar to those described in previous reports.^21^ Using this dexamethasone system, we identified the induction of amplitudes of 2 genes: Fxr1 and Ppat. Moreover, immunostaining demonstrated that PPAT was expressed in the nucleus and cytoplasm of the tubules, and FXR1 was expressed in the cytoplasm of the tubules. These results suggest that these 2 molecules are controlled by the renal peripheral clock and are potential candidates for blood pressure circadian rhythm formation.

Western blotting revealed that the protein expression of FXR1 was lower in the renal medulla of SHR rats than in that of WKY rats. The expression of PPAT in the renal medulla of WKY rats and SHR did not differ to a statistically significant extent. Fxr1 belongs to the same gene family as fragile X mental retardation gene 1 (Fmr1), which is considered the causative gene of fragile X syndrome. FXR1 is an RNA binding protein that interacts with FMR1 and FXR2. It is hypothesized that the cytoplasm and nucleus can be reciprocated because they have nuclear localization signals and nuclear export signals.^24^ In humans, the aberrant expression of Fxr1 in skeletal muscle has been shown to cause facioscapulohumeral muscular dystrophy.^25^ In this study, the Fxr1 expression was reduced in the kidneys of SHR and SHRSP rats and was expressed in tubular cytoplasm of mesodermal origin, which is the same as that of skeletal muscle, and may be involved in the proliferation and growth of tubular cells.

PPAT forms a donut-shaped homotetramer and becomes an inactivated homotetramer by binding purine nucleotides to affect the uric acid metabolism. In this study, the expression of PPAT was increased in the kidneys of SHR and SHRSP rats, and PPAT was localized in the cytoplasm and nucleus of tubular cells. Since uric acid has strong vascular toxicity and is an independent risk factor for arteriosclerosis. Thus, aberrant PPAT function might be associated with the pathophysiological features of SHR.

This study applied sequence and expression analyses and cultured cell experiments in a new attempt to narrow down the molecules that are related to blood pressure regulation and which are controlled by the biological clock. Furthermore, Fxr1 and Ppat are controlled by the biological clock and are related to blood pressure regulation. Further studies are necessary to measure and confirm the temporal variation of the Fxr1 and Ppat gene expression in the rat kidney. It is expected that the mechanisms of action of these molecules will be clarified. The control of these molecules is considered a target for the biological treatment of blood pressure fluctuation abnormalities.

**Figure 6.**
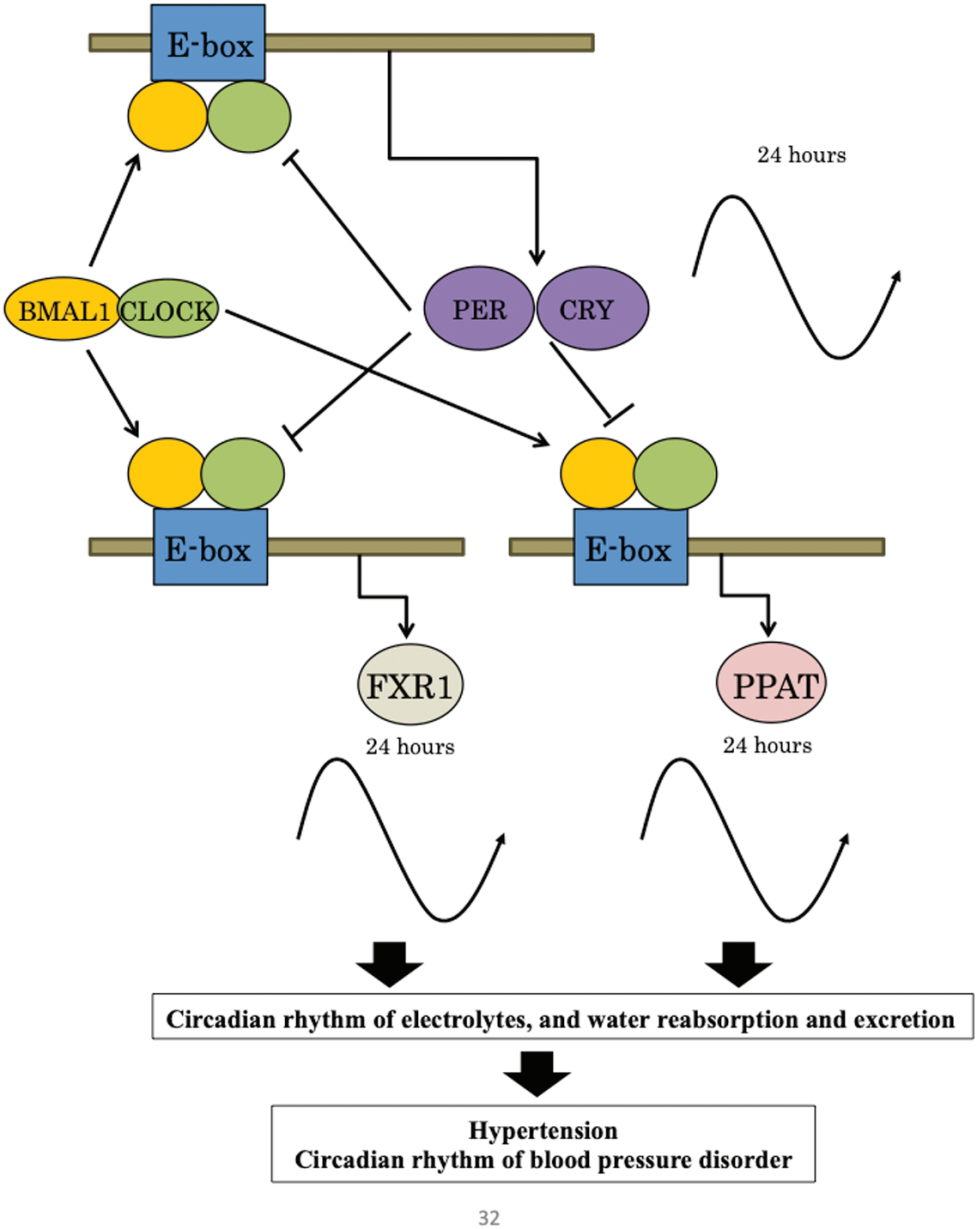
Hypothesized circadian feedback loop in the kidney of spontaneously hypertensive rats (SHRs). The following possible relationships were identified: PPAT and FXR1 with E-Box, and BMAL1 and CLOCK.

Based on the findings of the present study, a possible circadian feedback loop in the kidney from SHR is shown in Figure 6. PPAT and FXR1 have an EBOX in which BMAL1 and CLOCK bind to the promoter, suggesting that they be involved in the formation of circadian rhythm in tubules under the control of clock genes. It is considered that they are involved in the reabsorption and excretion of water and electrolytes in the tubules, and that they may affect hypertension and the circadian rhythm of blood pressure.

Thus, PPAT and FXR1 were considered molecules involved in the formation of blood pressure circadian rhythm in the kidney. With regard to the mechanisms of blood pressure regulation, these molecules are leading candidates for studies seeking to elucidate the mechanisms underlying the formation of blood pressure circadian rhythm.

## Supplementary Material

hpaa123_suppl_Supplementary_Informations

## References

[1] London AS Circadian biology: a 2.5 billion year old clock. Curr Biol 2012; 22:570–571. 10.1016/j.cub.2012.06.023 22835791

[2] Honma K, Honma S The SCN-independent clocks, methamphetamine and food restriction. Eur J Neurosci 2009; 30:1707–1717. 19878275 10.1111/j.1460-9568.2009.06976.x

[3] King DP, Zhao Y, Sangoram AM, Wilsbacher LD, Tanaka M, Antoch MP, Steeves TD, Vitaterna MH, Kornhauser JM, Lowrey PL, Turek FW, Takahashi JS Positional cloning of the mouse circadian clock gene. Cell 1997; 89:641–653. 9160755 10.1016/s0092-8674(00)80245-7 PMC3815553

[4] Ripperger JA, Schibler U Rhythmic CLOCK-BMAL1 binding to multiple E-box motifs drives circadian Dbp transcription and chromatin transitions. Nat Genet 2006; 38:369–374. 16474407 10.1038/ng1738

[5] Zhang EE, Kay SA Clocks not winding down: unravelling circadian networks. Nat Rev Mol Cell Biol 2010; 11:764–776. 20966970 10.1038/nrm2995

[6] Vollmers C, Gill S, DiTacchio L, Pulivarthy SR, Le HD, Panda S Time of feeding and the intrinsic circadian clock drive rhythms in hepatic gene expression. Proc Natl Acad Sci USA 2009; 106:21453–21458. 19940241 10.1073/pnas.0909591106 PMC2795502

[7] Kario K, Matsuo T, Kobayashi H, Imiya M, Matsuo M, Shimada K Nocturnal fall of blood pressure and silent cerebrovascular damage in elderly hypertensive patients. Advanced silent cerebrovascular damage in extreme dippers. Hypertension 1996; 27:130–135. 8591875 10.1161/01.hyp.27.1.130

[8] Curtis AM, Cheng Y, Kapoor S, Reilly D, Price TS, Fitzgerald GA Circadian variation of blood pressure and the vascular response to asynchronous stress. Proc Natl Acad Sci USA 2007; 104:3450–3455. 17360665 10.1073/pnas.0611680104 PMC1802007

[9] Zuber AM, Centeno G, Pradervand S, Nikolaeva S, Maquelin L, Cardinaux L, Bonny O, Firsov D Molecular clock is involved in predictive circadian adjustment of renal function. Proc Natl Acad Sci USA 2009; 106:16523–16528. 19805330 10.1073/pnas.0904890106 PMC2752602

[10] Dashti HS, Aslibekyan S, Scheer FA, Smith CE, Lamon-Fava S, Jacques P, Lai CQ, Tucker KL, Arnett DK, Ordovás JM Clock genes explain a large proportion of phenotypic variance in systolic blood pressure and this control is not modified by environmental temperature. Am J Hypertens 2016; 29:132–140. 26045533 10.1093/ajh/hpv082 PMC5863877

[11] Manchester RC The diurnal rhythm in water and mineral exchange. J Clin Invest 1933; 12:995–1008. 16694193 10.1172/JCI100569 PMC435957

[12] Dyer AR, Martin GJ, Burton WN, Levin M, Stamler J Blood pressure and diurnal variation in sodium, potassium, and water excretion. J Hum Hypertens 1998; 12:363–371. 9705037 10.1038/sj.jhh.1000601

[13] Gumz ML, Cheng KY, Lynch IJ, Stow LR, Greenlee MM, Cain BD, Wingo CS Regulation of αENaC expression by the circadian clock protein Period 1 in mpkCCD(c14) cells. Biochim Biophys Acta 2010; 1799:622–629. 20868778 10.1016/j.bbagrm.2010.09.003 PMC2975761

[14] Stow LR, Richards J, Cheng KY, Lynch IJ, Jeffers LA, Greenlee MM, Cain BD, Wingo CS, Gumz ML The circadian protein period 1 contributes to blood pressure control and coordinately regulates renal sodium transport genes. Hypertension 2012; 59:1151–1156. 22526258 10.1161/HYPERTENSIONAHA.112.190892 PMC3366275

[15] Munakata M, Imai Y, Minami N, Sasaki S, Ichijyo T, Yoshizawa M, Sekino H, Abe K, Yoshinaga K Cosinor analysis of changes in circadian blood pressure rhythm with aging in spontaneously hypertensive rats. Tohoku J Exp Med 1990; 161:55–64. 2396257 10.1620/tjem.161.55

[16] Tanaka S, Ueno T, Tsunemi A, Nagura C, Tahira K, Fukuda N, Soma M, Abe M The adrenal gland circadian clock exhibits a distinct phase advance in spontaneously hypertensive rats. Hypertens Res 2019; 42:165–173. 30464218 10.1038/s41440-018-0148-8

[17] Oster H, Challet E, Ott V, Arvat E, de Kloet ER, Dijk DJ, Lightman S, Vgontzas A, Cauter EV The functional and clinical significance of the 24-hour rhythm of circulating glucocorticoids. Endocr Rev 2017; 38:3–45. 27749086 10.1210/er.2015-1080 PMC5563520

[18] Nader N, Chrousos GP, Kino T Circadian rhythm transcription factor CLOCK regulates the transcriptional activity of the glucocorticoid receptor by acetylating its hinge region lysine cluster: potential physiological implications. FASEB J 2009; 23:1572–1583. 19141540 10.1096/fj.08-117697 PMC2669420

[19] Firsov D, Bonny O Circadian rhythms and the kidney. Nat Rev Nephrol 2018; 14:626–635. 30143787 10.1038/s41581-018-0048-9

[20] Cho H, Zhao X, Hatori M, Yu RT, Barish GD, Lam MT, Chong LW, DiTacchio L, Atkins AR, Glass CK, Liddle C, Auwerx J, Downes M, Panda S, Evans RM Regulation of circadian behaviour and metabolism by REV-ERB-α and REV-ERB-β. Nature 2012; 485:123–127. 22460952 10.1038/nature11048 PMC3367514

[21] Balsalobre A, Brown SA, Marcacci L, Tronche F, Kellendonk C, Reichardt HM, Schütz G, Schibler U Resetting of circadian time in peripheral tissues by glucocorticoid signaling. Science 2000; 289:2344–2347. 11009419 10.1126/science.289.5488.2344

[22] Balsalobre A, Damiola F, Schibler U A serum shock induces circadian gene expression in mammalian tissue culture cells. Cell 1998; 93:929–937. 9635423 10.1016/s0092-8674(00)81199-x

[23] Matsumura R, Node K, Akashi M Estimation methods for human circadian phase by use of peripheral tissues. Hypertens Res 2016; 39:623–627. 27334057 10.1038/hr.2016.68

[24] Adams-Cioaba MA, Guo Y, Bian C, Amaya MF, Lam R, Wasney GA, Vedadi M, Xu C, Min J Structural studies of the tandem Tudor domains of fragile X mental retardation related proteins FXR1 and FXR2. PLoS One 2010; 5:e13559. 21072162 10.1371/journal.pone.0013559 PMC2970552

[25] Davidovic L, Sacconi S, Bechara EG, Delplace S, Allegra M, Desnuelle C, Bardoni B Alteration of expression of muscle specific isoforms of the fragile X related protein 1 (FXR1P) in facioscapulohumeral muscular dystrophy patients. J Med Genet 2008; 45:679–685. 18628314 10.1136/jmg.2008.060541

