# Supplementary material for: Identification of Clock Genes Related to Hypertension in Kidney From Spontaneously Hypertensive Rats
Source: Am J Hypertens. 2020 Oct 14;33(12):1136–45. doi: 10.1093/ajh/hpaa123 (PMC7814221; doi:10.1093/ajh/hpaa123)
Supplement: hpaa123_suppl_Supplementary_Informations [file hpaa123_suppl_supplementary_informations.docx]

**Supplementary Informations**

**Identification of clock genes related to hypertension in kidney from spontaneously hypertensive rats**

Yusuke Murata^1^, Takahiro Ueno^1^, Sho Tanaka^1^, Hiroki Kobayashi^1^, Masahiro Okamura^1^, Seiichiro Hemmi^1^, Yoshinobu Fuke^1^, Yoshiaki Matsumoto^2^, Masanori Abe^1*^ & Noboru Fukuda^1,3*^

^1^Division of Nephrology, Hypertension and Endocrinology, Department of Internal Medicine, Nihon University School of Medicine, Tokyo, Japan

^2^Department of Clinical Pharmacokinetics, School of Pharmacy, Nihon University, Chiba, Japan

^3^Nihon University Research Center, Tokyo, Japan

**Supplemental Table 1.**

Primer list of TaqMan probes

| Per1 | Mm00501813_m1 |
| --- | --- |
| Bmal1 | Mm00500226_m1 |
| Cry1 | Mm00514392_m1 |
| Rev-erbα | Mm00520708_m1 |
| Npm1 | Mm02391781_g1 |
| Hnrnpa3 | Mm00817148_g1 |
| Tef | Mm00457513_m1 |

Primer list for SYBR Green method

| Tbl1x | forward (5' to 3') | AAG ATG AGC ATT ACC AGC GAC G |
| --- | --- | --- |
|  | reverse (5' to 3') | TGT GAC TTT CGA TCC CAA ACG T |
| Plagl1 | forward (5' to 3') | CTG ATG AGA CAC ATG GCC ACA C |
|  | reverse (5' to 3') | ATC GTC ACA CGC GTA GGA GAT C |
| Plbd1 | forward (5' to 3') | CTG GTC CCC TGA ATC GAA GAA |
|  | reverse (5' to 3') | CCC AGC CTG TGG TTT TAA TGG |
| Nptx1 | forward (5' to 3') | AGG CAG CAT CAG CTT TGA CAA T |
|  | reverse (5' to 3') | GCC GAC CCA TCA GGA AAC TTA |
| Fxr1 | forward (5' to 3') | ATA ATT GGC AAC CAG AAC CGC C |
|  | reverse (5' to 3') | TGG CTC TTG GTC ATT TGC TCT T |
| Bcl6 | forward (5' to 3') | ATG CTG ATG CCC CAT GAC AT |
|  | reverse (5' to 3') | GCA AAA GCT CTG CTC TCA CAC C |
| Ppat | forward (5' to 3') | GTC AGC AGC TGG CAA TTG AAG |
|  | reverse (5' to 3') | CCC ACA CTT TGT TGC ATA TCC C |
| Trim46 | forward (5' to 3') | AGC TGC ATA ACA GGA TTG CCC |
|  | reverse (5' to 3') | TCA ACA GCT TCA TCT CAC GCC |

**Supplemental Table 2.**

Search for genes with E-box in the promoter by GSEA


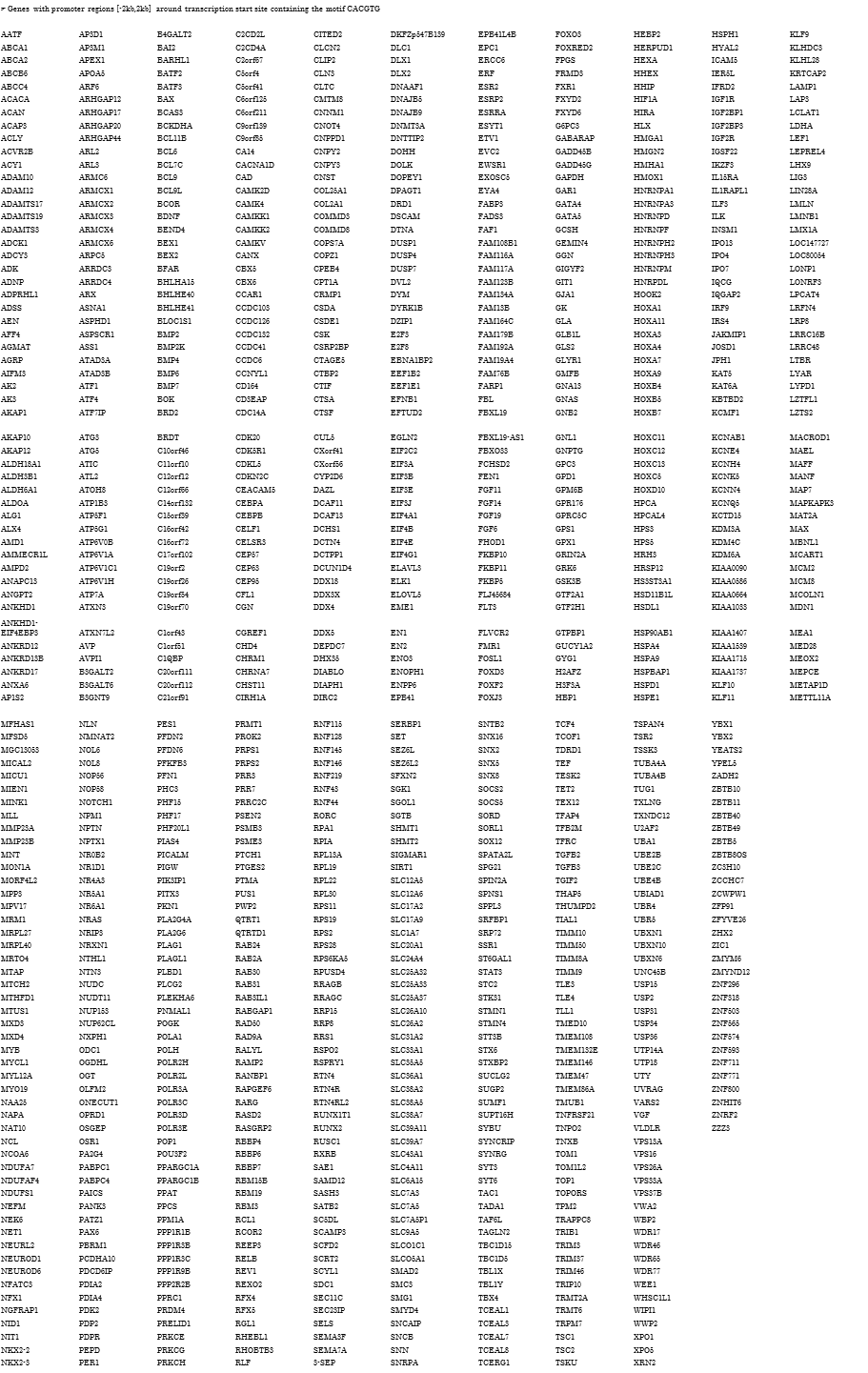


**Supplemental Table 3.**

Analysis of variance of clock genes and R^2^


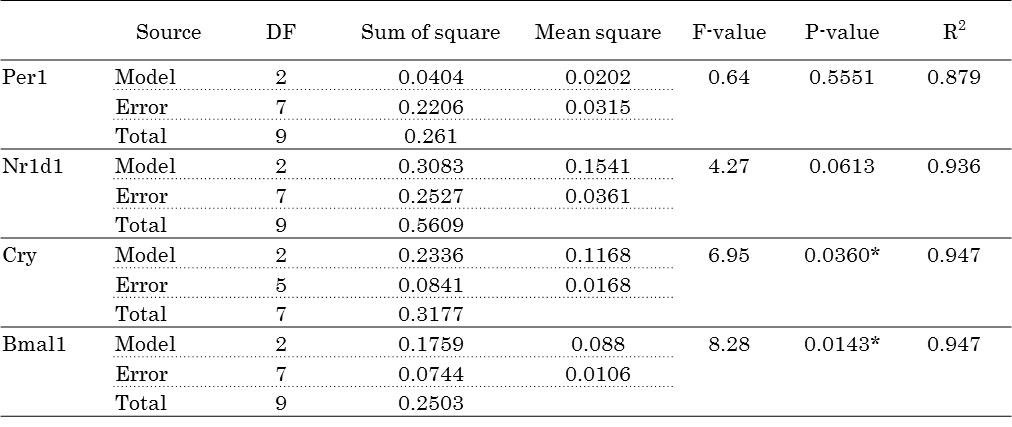


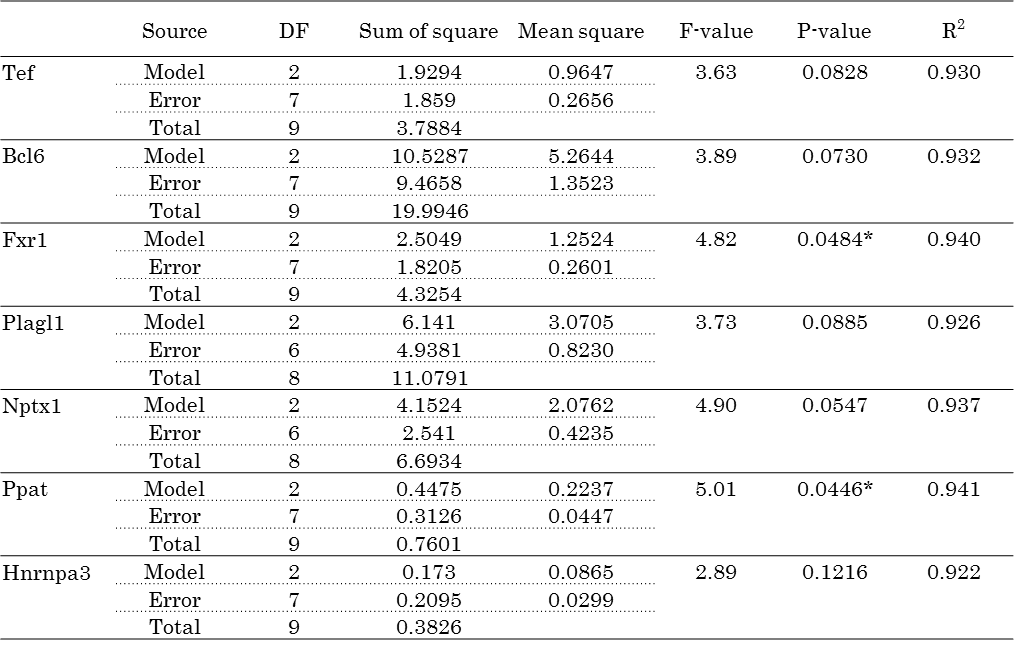


**Supplemental Figure 1.**

**
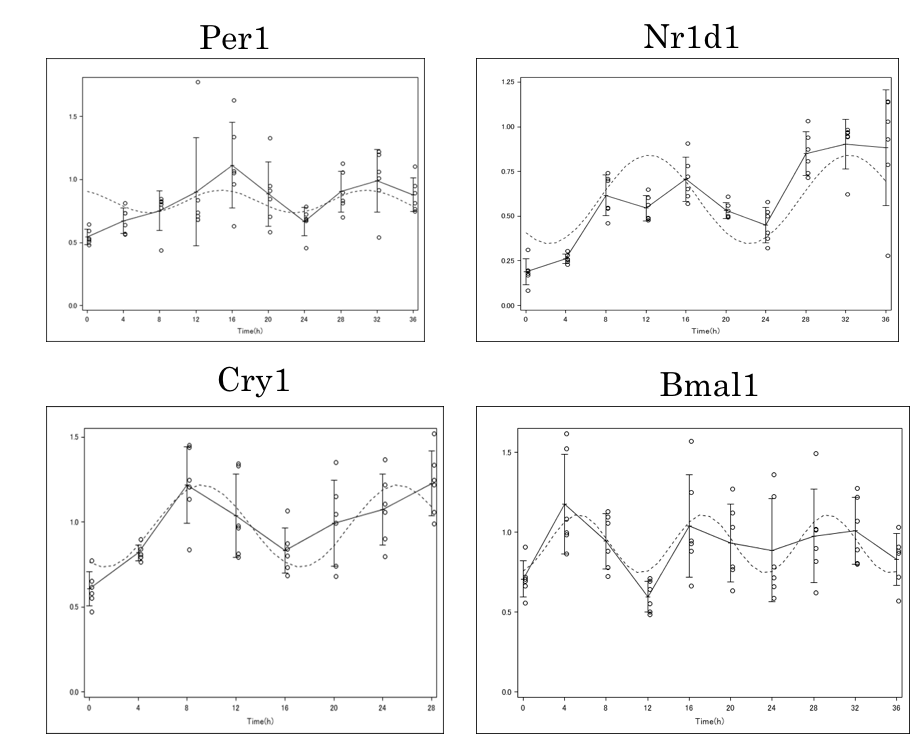
**

Periodic regression analysis of the clock gene mRNA expression. The periodicity of Per1, Bmal1, Nr1d1 and Cry1 mRNAs fluctuation after dexamethasone stimulation of TCMK-1 cells was analyzed by a multivariate regression analysis that found the mesh curve and amplitude, phase by optimizing the cosine curve by the least squares method, and their 95% confidence intervals were calculated using the single-coincidence method. The SAS (version 9.4) software program was used for the analysis, which was performed using the cosine method. Data are presented as the mean ± SEM (n=6).

**Supplemental Figure 2.**

**
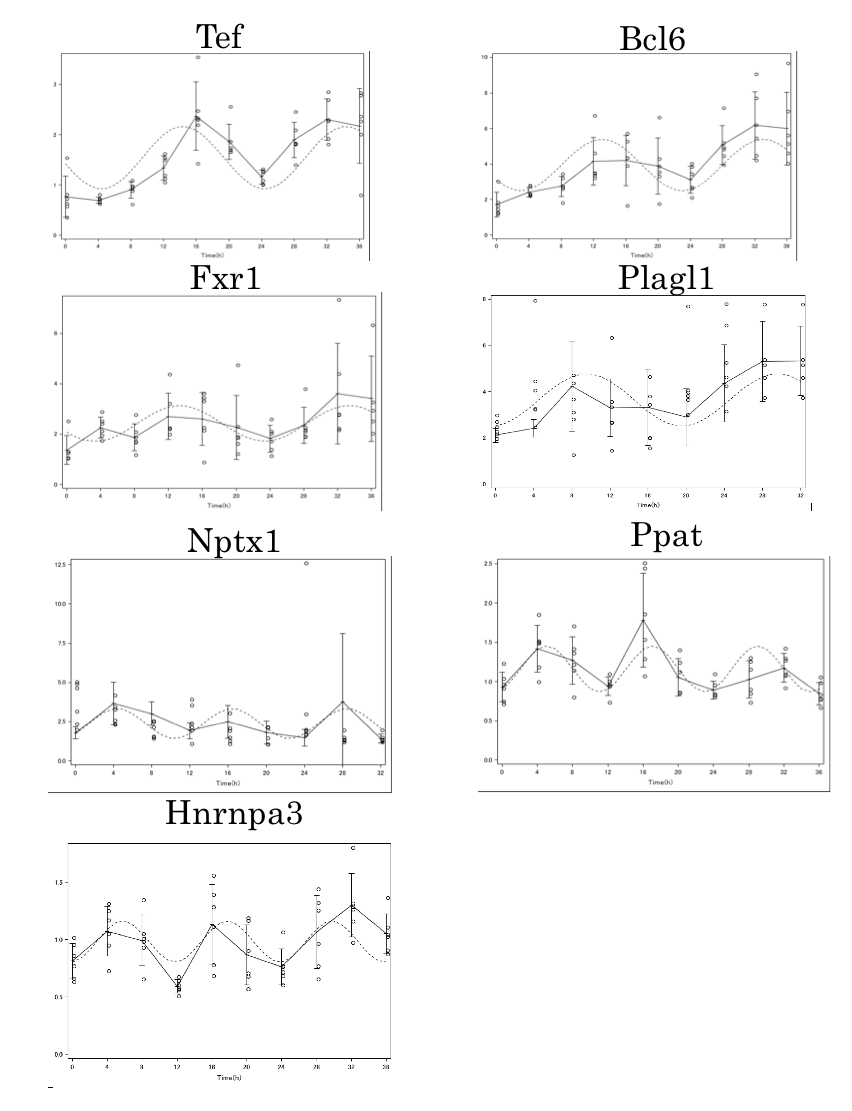
**

Periodic regression analysis of the clock gene mRNA expression. The periodicity of Per1, Bmal1, Nr1d1 and Cry1 mRNAs fluctuation after dexamethasone stimulation of TCMK-1 cells was analyzed by a multivariate regression analysis that found the mesh curve and amplitude, phase by optimizing the cosine curve by the least squares method, and their 95% confidence intervals were calculated using the single-coincidence method. The SAS (version 9.4) software program was used for the analysis, which was performed using the cosine method. Data are presented as the mean ± SEM (n=6).
